# Temporal Eating Patterns and Ultra-Processed Food Consumption Assessed from Mobile Food Records of Australian Adults

**DOI:** 10.3390/nu17203302

**Published:** 2025-10-21

**Authors:** Janelle D. Healy, Satvinder S. Dhaliwal, Christina M. Pollard, Amelia J. Harray, Lauren Blekkenhorst, Fengqing Zhu, Deborah A. Kerr

**Affiliations:** 1 Curtin School of Population Health, Curtin University, GPO Box U1987, Perth, WA 6845, Australia; janelle.healy@student.curtin.edu.au (J.D.H.); s.dhaliwal@curtin.edu.au (S.S.D.); c.pollard@curtin.edu.au (C.M.P.); 2 Office of the Provost, Singapore University of Social Sciences, 463 Clementi Road, Singapore 599494, Singapore; 3 Health & Social Sciences, Singapore Institute of Technology, 1 Punggol Coast Road, Singapore 828608, Singapore; 4 Obstetrics & Gynaecology Academic Clinical Program, Duke-NUS Medical School, National University of Singapore, 8 College Road, Singapore 169857, Singapore; 5 Curtin Medical Research Institute, Curtin University, Kent Street, GPO Box U1987, Perth, WA 6845, Australia; 6 Faculty of Health Sciences, enABLE Institute for Health Science, Curtin University, GPO Box U1987, Perth, WA 6845, Australia; 7 Medical School, The University of Western Australia, Verdun Street, Nedlands, WA 6009, Australia; amelia.harray@uwa.edu.au; 8 Childrens’ Diabetes Centre, The Kids Research Institute, Hospital Avenue, Nedlands, WA 6009, Australia; 9 Nutrition and Health Innovation Research Institute, School of Medical and Health Sciences, Edith Cowan University, Perth, WA 6027, Australia; l.blekkenhorst@ecu.edu.au; 10 Elmore Family School of Electrical and Computer Engineering, Purdue University, West Lafayette, IN 47907, USA; zhu0@purdue.edu

**Keywords:** temporal patterns, dietary assessment, ultra-processed foods, mobile food record, digital health, chrono-nutrition, dietary patterns

## Abstract

**Background/Objective:** Temporal eating patterns and ultra-processed food (UPF) consumption have independently been associated with obesity and non-communicable diseases. Little is known about the temporal patterns of UPF consumption, as data is challenging to collect. Temporal data can be extracted from mobile food records (mFRs). The aim of this study was to identify the temporal eating patterns of those consuming UPFs using an mFR. **Methods:** A combined sample of 243 young (18–30 years) and 148 older (>30 years) adults completed a 4-day mFR. The time of eating was extracted from the mFR image metadata. UPFs were identified using the NOVA food classification system. The proportion of total energy intake (EI) from UPFs was calculated hourly. Using chi-square tests, a day-of-the-week analysis compared weekends (Friday–Sunday) with weekdays (Monday–Thursday). A multivariate logistic regression of UPF EI terciles was conducted, expressed as odds ratios and 95% confidence intervals. **Results:** The proportion of total EI from UPFs was significantly different between younger adults (mean ± SD = 48.8 ± 15.6%) and older adults (36.1 ± 15.1%) (*p* < 0.001). Age-differentiated 24 h temporal eating pattern analysis found that younger adults had two distinct UPF EI peaks, with the highest at 8 pm, followed by 1 pm. Older adults followed a more conventional three-meal pattern with an additional peak at 7 am. Weekend UPF EI was higher than on weekdays for older adults (~560 kJ, *p* = 0.003), with no difference for younger adults. Multivariable logistic regression found no significant associations between UPF intake terciles and demographic variables (sex, BMI, education). **Conclusions:** The peak UPF EI occurred at conventional mealtimes, and UPFs accounted for a substantial proportion of energy intake, especially for younger adults. The timing of UPF EI provides important information for developing public health nutrition interventions.

## 1. Introduction

Temporal eating patterns, referring to “the timing, frequency and regularity of food intake” [1] (p. 2), are increasingly associated with dietary quality [2], metabolic changes, and health outcomes [3,4]. The time and day of the week can influence food choices; consequently, temporal eating patterns are an important area of research [2,4,5]. Capturing this information from dietary assessment methods such as dietary recalls and records is challenging, mostly because it relies on self-reported information. The temporal details of any eating occasion may be guided by meal names (e.g., breakfast), pre-structured mealtime estimates (e.g., 7:30 AM), or participants rounding times to the hour or half-hour [6,7]. Researchers concluded that Australians tend to eat at the conventional times of noon and 6 pm when they analysed self-reported eating occasion type and time in two 24 h dietary recalls [1]. Furthermore, inconsistent language in reporting meal timing and frequency in research findings has hampered temporal dietary assessment [4,6,8,9,10]. The nuanced details of temporal pattern analysis are often lost when summarised into inconsistent categories, for example, data-driven binary groups, such as early or late eaters [7,8], predetermined mealtime names [9], or weekends versus weekdays [11]. The limitations of the subjective recording of temporal data in conventional dietary recalls or food diaries have hampered temporal pattern analysis in informing dietary advice [5]. Researchers have suggested that temporal eating pattern evidence would benefit from time-stamped dietary intake records containing neutral eating occasion descriptions, such as those seen in image-based methods [7,9].

Identifying temporal eating patterns using image-based food records is an innovative approach to temporal dietary assessment. It contributes to important evidence on the timing of and variation in food intake associated with health outcomes. Image-based digital dietary assessment in the current study enables the use of temporal eating pattern data automatically captured with Technology Assisted Dietary Assessment (TADA), linked to a mobile food record (mFR) app [12,13,14]. The image metadata contained the date and time in hours and minutes, enabling an innovative analysis of dietary records to determine temporal eating patterns. Analysing image-based dietary records in the mFR app is an acceptable and accurate dietary energy intake assessment method [15] in both controlled [16,17] and community-dwelling adult studies [18,19]. The novel use of temporal data will provide greater insight into temporal dietary behaviours to inform practical dietary advice and guidance.

Contemporary eating patterns regularly include the daily consumption of packaged and highly processed convenience foods [20,21] with associated diet-related disease risk [22], independent of total energy intake (TEI) [23]. The food classification systems used to inform food selection guides and food-based dietary guidelines in many countries are predominantly based on the risks associated with the nutrient (sodium, sugars, saturated fat) and ingredient-centric (added sugar, salt, and fat) classifications of foods. Most of these foods meet the criteria for highly processed foods and contain added fats, sugar, and salt for increased palatability and shelf life [24]. The NOVA food classification system groups foods according to the extent and intent of industrial processing to assist in understanding the health risk associated with their consumption [25]. The four NOVA food classification groups are as follows: (1) nutrient-dense minimally processed (MP) whole foods, such as the edible parts of plants, animals, or fungi; (2) processed culinary ingredients derived from group 1 MP whole foods that are used in small amounts, added to MP foods to make them edible; (3) processed foods, which are most often a combination of MP and processed culinary ingredients with group 3’s processing intention to preserve the food or enhance palatability; and (4) ultra-processed food (UPF) formulations with cosmetic (e.g., colour) and industrial (e.g., emulsifier) additives used in food manufacturing to create hyper-palatable, shelf-stable products, typically energy-dense and low in beneficial nutrients, such as dietary fibre, protein, vitamins, and minerals [26,27]. The strength of the evidence of the adverse health impact of consuming UPFs is increasing [3,20,28,29,30,31]. The Australian Dietary Guidelines emphasise that an eating pattern comprising a wide variety of nutritious foods from the five food groups every day, consisting of predominantly minimally processed foods, is associated with optimal health [32]. Currently, there is no time-based component of dietary advice. The most recent Australian dietary survey shows that people are not meeting the recommendations [31,33] and therefore are not likely following the practical advice in the dietary guideline resources. Specifically, targeting dietary advice based on the times at and days on which people are more likely to eat UPFs may increase the salience and effectiveness of the advice and improve diet quality. This is the first study to investigate image-based dietary assessment using an mFR app to inform a temporal pattern analysis of UPF consumption over 24 h and on different days of the week.

The primary aim of this study was to identify two temporal patterns associated with UPF intake using the mFRs of Australian adults: (1) patterns over 24 h and (2) patterns for comparing differences between weekends (Friday–Sunday) and weekdays (Monday–Thursday). The secondary aim was to examine the associations between energy from UPF consumption and sociodemographic factors.

## 2. Materials and Methods

### 2.1. Study Characteristics

Analysis was conducted on the baseline demographic data and image-based mFRs from two samples of adults from Perth, Western Australia. The first sample of 243 young adults (18–30 years) was taken from a 6-month randomised controlled trial to improve nutrition behaviours using dietary feedback. The protocol and study outcomes have previously been published [19,34]. Briefly, adults were recruited from the Federal Electoral Roll. They were excluded if they could not complete the 6-month study, were undertaking extreme forms of exercise, were on a special diet, were currently studying or had previously studied nutrition, were pregnant or breastfeeding, could not attend the study centre to complete face-to-face assessments, or had any serious illnesses. The second sample (*n* = 148) was drawn from a 1-year randomised controlled trial (6 months of intervention and 6 months of follow-up) to investigate whether a tailored intervention using mobile technology can improve diet and physical activity behaviours leading to weight loss in adults (18–65 years) who are overweight or obese. The protocol has been previously published [35]. Briefly, adults were excluded based on serious illness or medical conditions, recent weight loss, medication use associated with weight management, pregnancy or current breastfeeding, current tobacco smoking, daily alcohol consumption greater than five standard drinks, prior or planned weight loss surgery, and the regular use of an activity monitor in the previous 12 months. There is currently no formal consensus on what constitutes young adulthood [36]. Those aged over 30 years were selected to align with the age groups reported in the Nutrient Reference Values [37] (e.g., energy intake). The Nutrient Reference Values inform the Australian Dietary Guidelines [32]. Both trials were registered with the Australian New Zealand Clinical Trials Registry (reference ACTRN12612000250831, registration date 29 February 2012 and ACTRN12617000554369, registration date 20 April 2017), and the projects were approved by the Curtin University Human Research Ethics Committee (HR181/2011, approval date 8 February 2011 and HR61/2016, approval date 6 April 2016).

### 2.2. Dietary Analysis

Adults in both samples were asked to capture before-and-after images of all meals, snacks, and beverages consumed over four consecutive days, including one weekend day. The images were captured on their mobile device using the mFR app (TADA3.3.1.ipa and 1.0.chat2i) linked to the Technology Assisted Dietary Assessment (TADA) system [12,13,14]. The days of the week were grouped into weekends (Friday–Sunday) and weekdays (Monday–Thursday). Friday was included as a weekend day as the eating patterns on Fridays have previously been found to resemble those on other weekend days (Saturday and Sunday) [38]. The time of eating in hours and minutes was taken from the mFR metadata. A dietitian reviewed and confirmed the contents of the images.

A dietitian trained analyst identified the food and beverage items and portions eaten from the mFR images. The weight or household measure was recorded using nutrition analysis software (Foodworks 9, Xyris Pty Ltd., Brisbane, Australia), which was linked to the Australian Food Composition Database (AUSNUT 2011-13) [39] to estimate the energy contribution. The foods were matched to the published NOVA food classification [40], according to the definition of NOVA groups by the Food and Agriculture Organization of the United Nations [41]. We defined an eating occasion to encompass any ingestive event where the adults had captured an image, regardless of the nutrient contribution to the overall daily energy intake [2,9]. Removing a measure of energy requirement in the definition of an eating occasion considers emerging evidence of the health impact of non-nutritive additives in UPFs [20,42,43]. A food record day was included in the analysis if at least one image was recorded over the day. Reported eating occasions without images were excluded from the analysis (157 from younger adults (18–30 years), 26 from older adults (>30 years)), as the temporal data were not objectively reported. A daily energy intake (EI) average of less than 2000 kJ was set as the minimum plausible EI [44]. Based on these criteria, no records were excluded. UPF eating frequency was defined as the number of UPFs consumed over the day [28]. Day-of-the-week analysis compared UPF EI and the number of UPF items eaten over the weekend (Friday–Sunday) and weekdays (Monday–Thursday) [38].

### 2.3. Temporal Pattern Statistical Analysis

Data analysis was conducted using SPSS 28 (IBM SPSS Statistics), with statistical significance determined at *p*-values greater than or equal to 0.05. A non-parametric Sign test, independent sample t-tests (continuous variables), and chi-square tests (categorical variables) were used to compare studies and tercile groups within the studies. A multifactorial logistic regression analysis was conducted where appropriate.

The mean daily TEI and UPF EI were calculated as the average over the recorded days from the mFRs using the AUS-NUT 2011-13 database [39]. The eating time (hour and minutes) was extracted from the mFR food image time-stamp. The proportion of EI from UPFs and TEI were summed per hour for each study. Microsoft Excel [45] was used to calculate and graph the proportion of energy intake (EI) from UPFs and total energy intake (TEI), per hour.

SPSS functionality was used to determine each group’s UPF EI tercile cut-off values. As study-group-specific and time-of-day-specific differences were identified from our analyses, a separate multivariable logistic regression for each group was conducted, expressed as odds ratios and 95% confidence intervals.

The date-stamps of the images of the food records were converted to days of the week using an Excel function (weekend eating occasions = 8094 (45%); weekday eating occasions = 9886 (55%)). Using Excel, each person’s daily food record was used to aggregate per person by weekends, defined as Friday to Sunday, and weekdays, defined as Monday to Thursday [38]. Foods were classified using the NOVA classification [40,41], and the frequency or number of food records in each food category was summed [28]. The mean EI for each subject was calculated for each of the four NOVA groups during weekdays and weekends.

## 3. Results

### 3.1. Demographics

Table 1 presents the demographic characteristics of the two groups. The proportion of TEI from UPFs was greater for younger adults compared with older adults (*p* < 0.001; mean 48.8 ± SD 15.59%, 36.1 ± 15.10%, respectively). The younger adult group (18–30 years) was observed to have a wider range of energy intake from UPFs (5–92% of TEI) than older adults (>30 years) (5–79% of TEI).

The TEI and macronutrient proportions of TEI were similar between both groups and were within the Acceptable Macronutrient Distribution Ranges [37] (younger adults up: TEI 7333 kJ ± 2370 kJ/d, protein 17%, carbohydrate 43%, fat 35%; older adults group: TEI 7473 ± 1864 kJ/d, protein 18%, carbohydrate 39%, fat 37%). Multivariable logistic regressions were conducted separately for each group. No demographic variables (sex, body mass index (BMI), education) were significantly associated with being in the highest UPF EI tercile group compared to the lowest tercile group. As such, the detailed regression outputs are not shown in tables. Within each group, BMI was not associated with the tercile group according to the Spearman Rank Correlation (*p* > 0.1).

### 3.2. Age-Differentiated Comparison of 24 h Temporal Pattern of UPF Intake

**Figure 1:**
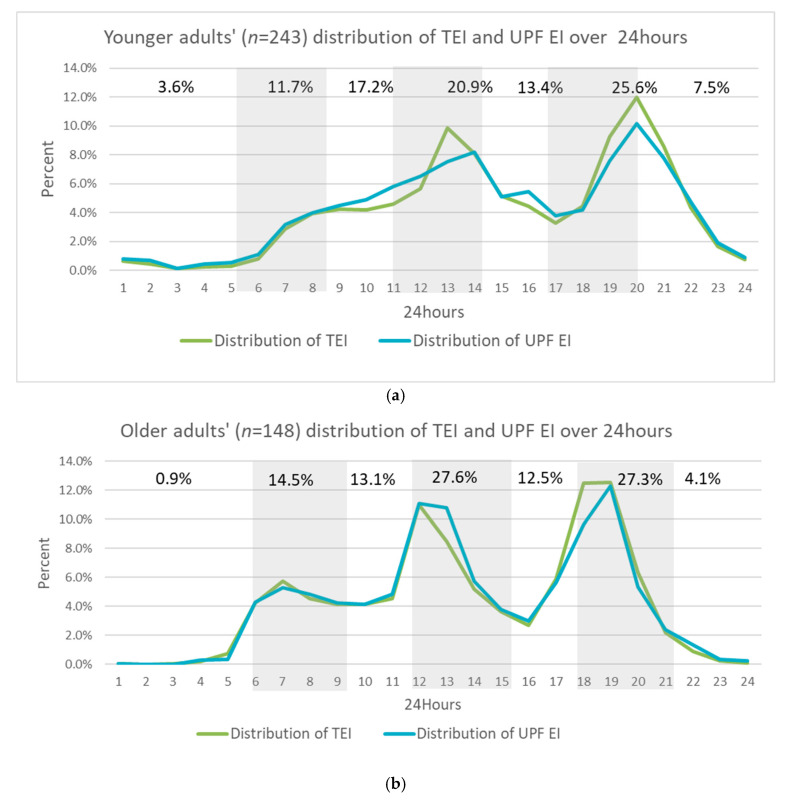
Intake distribution over 24 h of hourly proportion of TEI and UPF EI in (**a**) younger (18–30) and (**b**) older (over 30 years) Australian adults. Three-hour cumulative UPF EI proportion in text with shaded areas indicating conventional mealtimes.

There was a difference in the 24 h pattern of the proportion of UPF energy intake as a proportion of TEI between the two groups (*p* = 0.026). The temporal pattern of the proportion of UPF EI over 24 h between the two groups is plotted in Figure 1. The graph shows three distinct peaks of UPF EI for older adults at conventional mealtimes (breakfast, lunch, and dinner), with two peaks for younger adults at lunch and dinner times. The younger adults’ UPF temporal pattern showed a morning grazing pattern until 2 pm with an additional small peak in the proportion of UPF EI in the midafternoon (4 pm). The peak UPF EIs at conventional mealtimes were higher in older adults than in younger adults (Figure 1) and accounted for 58.2% and 69.4% of TEI in younger and older adults, respectively. By noon, younger adults had consumed 32.4% of TEI from UPFs, compared to 28.5% in older adults.

### 3.3. Temporal Patterns of Weekends and Weekdays

Table 2 shows that younger adults consistently obtained more energy from UPFs and less from minimally processed whole foods overall. The difference in UPF EI between weekends and weekdays in younger adults showed little variation (42.4 kJ, *p* = 0.808). Older adults recorded an increased energy contribution from UPFs on weekends (557.7 95% CI 199.7–915.7 kJ, *p* = 0.003) and a reduction in that from minimally processed foods (607.2 kJ, 95% CI −852.3–−362.0, *p* < 0.001).

A comparison of the eating frequency of UPFs for weekends versus weekdays was undertaken. mFR data were recorded on weekend days (*n* = 8094, 45%). The average number of UPF items consumed per person was 4.0 in both groups and did not change over the day of the week.

### 3.4. Most Frequent UPFs Consumed

The average number of UPFs consumed over the food record days was 4.0 per day for both younger and older adults and was the same across the days of the week. The five most frequently consumed UPFs are shown in Table 3, with individual items sorted into the Australian Dietary Guidelines’ food groups [30] with the discretionary food group further divided into snack or ready meals. For example, grain-based foods were combined, including bread rolls, loaves, and crackers. Table 3 shows the percentage of adults who recorded the UPF group and the frequency of eating these foods per person who consumed them. More younger adults consumed a greater variety of UPF items compared to older adults, except for bread-based foods. The most common UPFs eaten per day were bread-based foods (27% of older adults consumed on average four items per day; 17% of younger adults consumed on average two items per day), pizza as a ready-meal option (8% of older adults consumed on average one item per day; 12% of young adults consumed two items on average per day), and hot chips for savoury snacks (one item per day was consumed by 5% of older adults and 7% of younger adults).

## 4. Discussion

This study indicates that older adults displace energy from minimally processed foods with UPFs on weekends, without increasing the number of eating occasions. There was an age-differentiated pattern of UPF energy intake, though UPFs were eaten at all eating occasions for both the younger and older groups. Older adults ate UPFs in a conventional eating pattern of three meals daily and with more EI from UPFs on the weekends. Younger adults had a grazing TEI temporal eating pattern, including eating UPFs between 6 am and 2 pm, with a main peak at the conventional dinner eating occasion at 8 pm, after a secondary peak in the proportion of EI from UPFs at 4 pm. This secondary peak was not seen in older adults and may reflect afternoon snacking on UPFs, 2 h after the lunchtime peak in younger adults. Additionally, the analyses found that younger adults did not vary their food choices over the week. Younger adults obtained less energy from minimally processed whole foods and more from UPFs than older adults. These results concur with a meta-analysis that found that the adult consumers with the highest intake of UPFs in Australia are 19- to 30-year-olds [31]. The peak energy intake from UPFs in older adults was the highest between 6 pm and 9 pm, followed by 12 pm and 3 pm. This age-differentiated temporal pattern of UPF intake is consistent with reported conventional eating times for older adults and an overall grazing eating pattern of TEI in younger Australians [1]. This demonstrates the ubiquitousness of UPF consumption throughout the day, particularly at historically designated mealtimes. This suggests that specific timing-related messages for reducing UPF consumption may be warranted.

The novelty of this study lies in examining the temporal eating patterns of those consuming UPFs using image-based dietary assessment. Previous studies have conducted temporal analyses of overall food choices using the 24 h recall data from the 2011–2012 Australian Health survey [1,7,47,48]. These studies identified eating frequency and sociodemographic differences between the eating patterns of those consuming foods in general [1]. However, the analysis extracted temporal data from self-reported eating time and selection from predetermined eating occasion names (e.g., breakfast, lunch). Metadata from image-based dietary assessment in our study enabled automated, low-burden temporal data collection, overcoming the challenges of recall or predetermined mealtimes. Exploring the temporal eating patterns of those consuming UPFs adds practical UPF exposure details to research on the health impact of eating UPFs. A review by Elizabeth (2020) [3] found inconsistent reporting of UPF exposure, which limits meta-analyses of evidence. Examples included the percentage of energy intake, eating frequency, servings per day, and the ratio of UPF intake to minimally processed or processed food intake [49]. Meanwhile, we identified that every eating occasion is a potential opportunity for intervention, and weekend mealtimes could be an age-differentiated nutrition intervention target for Australians over 30 years old.

This is the first study to indicate that older adults displace energy from minimally processed foods with UPFs on weekends, without increasing the number of eating occasions. Recording an average of 4.0 UPFs consumed per day in this study is consistent with an investigation of the United States of America NHANES III 1988–1994 data [28], though the contribution to EI is lower in Australia (United States of America’s 35–65% versus Australia’s 39–42%) [50]. However, the proportion of daily energy intake from UPFs in the current study was higher on weekends for older adults. The current results concur with a US study that found a higher diet quality on weekdays [11]. The researchers proposed a Structured Day Hypothesis [51] whereby the unstructured nature of weekend activities, including eating, results in poorer diet quality. The time of day and the day of the week are cues or heuristics for UPF choices, and this information can be used to tailor dietary advice. Targeting dietary messaging based on when UPFs are eaten is warranted to reduce the high proportion of EI from UPFs of TEI in this study and the others mentioned above to under the 15% UPF EI threshold, associated with a reduced risk of living with overweight and obesity [52]. Using common heuristic rules, such as the time of day, to build the dietary advice frame [53] disrupts the food choice decision. These temporal results show evidence for contemporary tailored dietary advice incorporating age, eating time, and the day of the week. Incorporating a different approach to dietary messaging may help in starting to address the problem of high UPF consumption and help prevent avoidable non-communicable diet-related diseases [33].

Caution may be needed when adopting new food classification systems for dietary advice. The current study shows that some of the foods that were commonly consumed in high amounts were UPFs, but some are also recommended in Australian dietary guidance. Commercially produced bread was the most consumed UPF in both groups. The fortification of bread flour with thiamin and folate in Australia is a public health initiative. Commercially manufactured breads also contain cosmetic ingredients to keep them “supersoft”, protect them from mould, and extend their shelf life. The processing of commercially manufactured bread categorises it as a UPF, and broad Australian dietary recommendations classify it as a nutritious food. The need for specific messaging regarding UPFs that are fortified staple foods to address a public health need requires further investigation. Seven countries have specifically embraced the UPF concept in their national dietary recommendations already (Belgium, Brazil, Ecuador, Israel, Maldives, Peru, and Uruguay) [54].

Confidently classifying foods into the NOVA food groups requires additional information beyond that usually collected in food records. Researchers found [25] that interview-based methods that captured details, such as novel ingredients and whether the food was handmade or commercially made, increased confidence in classifying NOVA food groups. Image-based methods may capture the ingredients and preparation information in a low-burden, scalable manner, enabling a dietary assessment to be conducted against the NOVA food classification system [25]. UPF classification details are already being incorporated into the increasingly popular computerised 24 h recalls in population dietary surveillance [55]. Further temporal eating pattern research could harness image-based dietary assessment and additional contextual information, such as the location and duration of eating occasions, to inform dietary recommendations [56]. Incorporating temporal and contextual cues and heuristics in programmes, interventions, and dietary messaging may increase the salience of messages. Technology is being tested in some population groups to deliver web-based automated two-way messages in real time in response to dietary recalls [57]. Furthering the use of the mFR app, image-based dietary records, and real-time messages to increase the awareness of when UPFs are eaten can increase the salience or connection to dietary behaviour messages [58]. Strengthening temporal eating pattern research methods with the use of image-based methods may provide more targeted dietary advice considering the UPF temporary eating pattern. Investigating the effectiveness of practical and salient temporal UPF advice is the next step in developing dietary advice.

### Strengths and Limitations

A key strength of this study is the use of low-burden image-based mobile food records to capture eating times and days. In dietary studies and clinical practice, time- and date-stamped food records improve temporal eating pattern analysis data quality in dietary assessment. Without predefined mealtimes or eating occasions, images can define eating occasions, reflecting participants’ eating patterns without cultural labels [2]. The images remove the burden of the self-reported time of eating and inconsistent definitions of eating occasions [4,9,25]. This study also used the existing NOVA classification of the AUSNUT 2011-12 food database [40]. This provided a consistent classification of the Australian food database with other research, despite the identified limitations in the retrospective application of the NOVA classification system [25].

This analysis was conducted on multiple-day records, which can reflect usual eating patterns to account for unusual eating days and reduce some biases compared to 24 h food records [59]. However, reactivity bias in image-based dietary records may still occur [17]. These results may also be the minimum peaks of UPF temporal eating patterns over 24 h and across days of the week, as eating occasion data were excluded if there was no time-stamped image. Conducting larger-scale investigations is needed to confirm and extend the findings from these two nutrition-focused randomised controlled trials with a limited sample size of participants.

## 5. Conclusions

This study found age-differentiated temporal eating patterns in UPF consumption. Over 24 h, younger adults consumed UPFs in a grazing pattern, which did not change between weekdays and weekends. Older adults ate UPFs at conventional eating times with peak increases at breakfast, lunch, and dinner. Weekends were a cue or heuristic for UPF intake, as this was when older adults were more likely to include UPF choices. UPFs are pervasive in contemporary eating patterns; we found they are eaten in all meals and snacks. The use of image-based dietary records is a low-burden method for determining temporal eating patterns. With instructions to include ingredients and methods of manufacture in the food record, image-based dietary assessment can further the evidence for UPF temporal patterns to inform dietary advice. Contemporary dietary messaging could consider the emerging evidence of temporal eating patterns regarding the types, forms, and amounts of food consumed. Temporal eating pattern studies using low-burden image-based dietary assessment can further explore the heuristics or triggers for the consumption of UPF. Future research could develop and test temporal eating messages in larger populations and assess the effectiveness of dietary messages tailored to different age groups. Targeting the times at and days on which people are more likely to consume ultra-processed foods may increase the likelihood of dietary behaviour change and improved health.

## Figures and Tables

**Table 1:** Demographic characteristics of younger (18–30 years) and older (over 30 years) adults in temporal dietary pattern investigations of 4-day average energy intake from UPFs, over 24 h.

		Younger (*n* = 243)	Older (*n* = 148) ^1^
		Mean ± SD	Mean ± SD
Age (years)		24.3 ± 3.4	49.2 ± 9.7
BMI ^2^ kg/m^2^		24.3 ± 5.4	31.4 ± 3.9
Proportion of TEI from UPFs (%)		48.8 ± 15.59	36.1 ± 15.10
Range of proportion of TEI from UPFs (%)		5–92%	5–79%
		N (%)	N (%)
Sex			
	Male	82 (33.7%)	49 (33.3%)
	Female	161 (66.2%)	98 (66.7%)
BMI ^2^ category (%)			
	Healthy weight	166 (68.3%)	0 (0%)
	Overweight (≥25 kg/m^2^)	47 (19.3%)	63 (43.1%)
	Obese (≥30 kg/m^2^)	30 (12.3%)	83 (56.8%)
Ethnicity			
	Caucasian	187 (76.9%)	130 (88.4%)
	Aboriginal	4 (1.6%)	0 (0%)
	Asian	41 (16.9%)	9 (6.1%)
	Black African	1 (0.4)	2 (1.4%)
	Mixed	10 (4.1%)	6 (4.1%)
Education			
	Year 10, 11, or 12	86 (35.4%)	26 (17.7%)
	Trade or diploma	59 (24.3%)	34 (23.8%)
	University degree or higher	98 (40.3%)	87 (59.2%)

^1^ Demographic information was incomplete for one adult included in mFR analysis. ^2^ BMI is abbreviation for body mass index [46].

**Table 2:** Temporal pattern of mean energy per day, comparing weekend (Friday–Sunday) and weekday (Monday–Thursday) intake for younger (18–30 years) and older (over 30 years) adults.

	NOVA ^1^	Weekend (Mean ± SD kJ/d)	Weekday (Mean kJ/d)	Weekend–Weekday Difference (Mean kJ/d)	95% CI	*p*-Value ^2^
Younger (*n* = 243)	MP	2014.1 ± 1515.3	2211.7 ± 1496.8	−197.6	−440.2–44.9	0.110
	PCI	348.5 ± 216.0	281.1 ± 199.8	67.4	2.7–132.1	0.041
	P	2283.0 ± 1592.5	2284.7 ± 1454.8	−1.7	−265.5–262.1	0.990
	UPF	3579.5 ± 2072.9	3537.1 ± 1995.7	42.4	−301.1–385.8	0.808
Older (*n* = 148)	MP	2341.9 ± 1313.1	2949.1 ± 1286.5	−607.2	−852.3–−362.0	<0.001
	PCI	365.0 ± 382.1	433.4 ± 452.2	−68.4	−184.2–47.3	0.241
	P	2275.6 ± 1523.8	2224.4 ± 1298.9	51.2	−265.2–367.6	0.749
	UPF	3242.1 ± 1777.0	2684.4 ± 1677.5	557.7	199.7–915.7	0.003

^1^ MP, minimally processed foods in NOVA group 1; PCI, processed culinary ingredients in NOVA group 2; P, processed foods in NOVA group 3; UPF, ultra-processed foods in NOVA group 4. ^2^ *p*-value of difference between weekend and weekday for each age-differentiated group.

**Table 3:** Most frequent UPFs eaten by younger and older adults and temporal dietary pattern investigations of average energy intake from UPFs, over 24 h.

Younger (*n* = 243)				Older (*n* = 148)			
Summary of food items	Percent	Freq per person	Examples	Summary of food items	Percent	Freq per person	Examples
Bread and bread-based foods *	17%	2	White and wholemeal bread rolls, loaves, and fruit bread	Bread and bread-based foods	27%	4	White bread rolls and loaves, wholemeal loaves
Beverages (2% had alcohol)	17%	2	Cola-based soft drink, coffee	Sweet baked goods (biscuits and cakes)	11%	1	Plain biscuits, shortbread, cakes, and muffins
Ready meals (e.g., pizza)	12%	2	Pizza, hamburgers/wraps	Ready meals (e.g., pizza)	8%	1	Pizza
Sweet biscuits/cakes	11%	1	Muesli bars, plain sweet biscuits	Lollies, chocolate	7%	1	Chocolate, fruit chews
Breakfast cereal	8%	1	Mixed grain flaked or extruded cereal	Breakfast cereal	6%	1	Whole-wheat biscuits (e.g., weetbix)
Savoury snacks (e.g., hot chips)	7%	1	Chips, wedges, hash browns	Savoury snacks (e.g., hot chips)	5%	1	Hot chips

* Bread and bread-based foods and grain-based foods, including bread rolls, loaves, and crackers, were combined.

## Data Availability

The data presented in this study is available on request from the corresponding author.

## Abbreviations

The following abbreviations are used in this manuscript:

UPF	Ultra-processed food
EI	Energy intake
TEI	Total energy intake
mFR	Mobile food record
DG	Dietary guideline
